# Adaptive plasticity of auxetic Kirigami hydrogel fabricated from anisotropic swelling of cellulose nanofiber film

**DOI:** 10.1080/14686996.2024.2331959

**Published:** 2024-03-20

**Authors:** Daisuke Nakagawa, Itsuo Hanasaki

**Affiliations:** Institute of Engineering, Tokyo University of Agriculture and Technology, Koganei-shi, Japan

**Keywords:** Nanopaper, mechanical metamaterial, soft matter, mechanics of materials, soft robotics

## Abstract

Hydrogels are flexible materials that typically accommodate elongation with positive Poisson’s ratios. Auxetic property, i.e., the negative Poisson’s ratio, of elastic materials can be macroscopically implemented by the structural design of the continuum. We realize it without mold for hydrogel made of cellulose nanofibers (CNFs). The complex structural design of auxetic Kirigami is first implemented on the dry CNF film, i.e., so-called nanopaper, by laser processing, and the CNF hydrogel is formed by dipping the film in liquid water. The CNF films show anisotropic swelling where drastic volumetric change mainly originates from increase in the thickness. This anisotropy makes the design and fabrication of the emergent Kirigami hydrogel straightforward. We characterize the flexibility of this mechanical metamaterial made of hydrogel by cyclic tensile loading starting from the initial end-to-end distance of dry sample. The tensile load at the maximum strain decreases with the increasing number of cycles. Furthermore, the necessary work up to the maximum strain even decreases to the negative value, while the work of restoration to the original end-to-end distance increases from the negative value to the positive. The equilibrium strain where the force changes the sign increases to reach a plateau. This plastic deformation due to the cyclic loading can be regarded as the adaptive response without fracture to the applied dynamic loading input.

## IMPACT STATEMENT

Highly anisotropic swelling-based gelation of dry film of cellulose nanofibers enables development of emergent hydrogel Kirigami with auxetic behavior. Furthermore, adaptive response to the iterative loading condition is found.

## GRAPHICAL ABSTRACT

**Figure:**
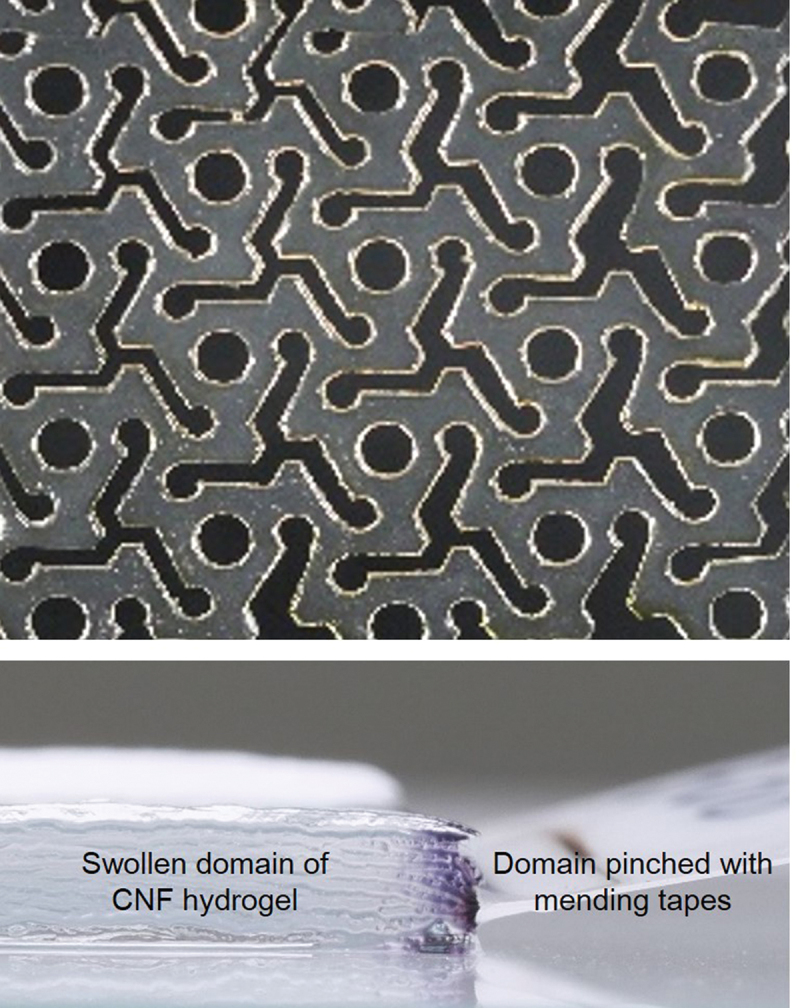

## 1. Introduction

Materials for substantial deformation without fracture are employed in systems with mechanical functionalities ranging from flexible devices [1–4] to soft robotics [5–8]. Elastomers such as silicone rubbers are flexible against tensile loading as well as bending. On the other hand, typical papers are stiff against tensile loading while flexible against bending [9]. This difference is related to the design of mechanical metamaterials [10]. Auxetic behaviors, i.e., negative Poisson’s ratio, are realized with in-plane deformation typically by structures with sufficient thickness [11–23]. Flexibility of elongation for paper-like sheet materials is realized by Kirigami structures [24–32]. Origami and Kirigami structures are most typically implemented on the sheet structures where thickness is sufficiently small [33,34]. They are designed to show out-of-plane deformation [30–32], and thereby elongation as a whole structure is attained by the combination of bending. This makes contrast with the mechanical metamaterials with thick elastomers mentioned above based on the in-plane deformation.

While the stiffness against tensile loading sometimes appears to be a drawback to be compensated by the Kirigami or Origami design for soft and flexible applications, it is an advantage when implementing electronic elements without tensile flexibility. Furthermore, the paper-based materials have advantages of environmental friendliness. In particular, film structures made of cellulose nanofibers (CNFs) [35] which is called ‘nanopapers’ [36–39] have been shown to be useful for substrates of flexible devices [39–41]. The nanopapers are promising for degradable electronics and green electronics [2,42]. The flexible substrates are often applied to wearable devices [3,43,44] subject to repeated deformation. Then, the mechanical resilience [32] is also an important characteristic to be explored. The importance of mechanical resilience is not limited to paper-based materials but it holds for various applications of flexible devices and soft robotics. In our previous study, we had pointed out this importance and defined the mechanical resilience by the residual strain after iterated elongation of the whole structure for mechanical metamaterial. Furthermore, we proposed a design principle of stress defocusing to enhance mechanical resilience of Kirigami metamaterial [32].

The nanopapers made of CNFs have a distinctive characteristic to form hydrogel when in contact with liquid water [45]. The CNF hydrogels can easily be possessed with thicknesses comparable to the order of other dimensions in a specimen in contrast to the dry nanopaper. Therefore, it is more feasible to design the functionality of mechanical metamaterials for in-plane deformation such as auxetic behavior. The swelling-based gelation is similar to the inverse process of nanopaper formation from drying aqueous CNF dispersion which also undergoes gelation before drying up [46,47]. Since the nanopaper formation is a process to form collective structure of fibers, the hydrogel is expected to be of less definite structure compared to the nanopapers. The reformation of bonds between the fibers might be easier. Therefore, the plasticity of CNF hydrogels might be useful for adaptive characteristics. In contrast to that the resilience is intended for restoration of the original state, the adaptive plasticity is intended for transition to other states from the original without fracture. Today, the adaptive material characteristics are attracting more attention [48,49] as part of the paradigm of intelligent materials [50–52].

In this study, we demonstrate the emergent auxetic Kirigami hydrogel made of CNFs and examine the response to the cyclic loading. The cyclic tensile loading corresponds to the incessant variation of end-to-end distance. Therefore, the loading condition does not have objective distinction of the unique and fundamental end-to-end distance, while the maximum and minimum end-to-end distances are special in terms of discontinuity in the sign of strain rate. In other words, this cyclic loading can be regarded either as tensile loading from minimum end-to-end distance or compression from maximum end-to-end distance. Unless the original state is clearly defined, the decrease of end-to-end distance is a compression, and the increase of it is an elongation. The results indicate that the samples reach another state from initial condition, which is confirmed from the strain with vanishing load and work necessary for a single cycle of tension and compression. This is important from the viewpoint of adaptive behavior of materials and memory of them [53] toward intelligent system applications.

## 2. Methods

### 2.1. Fabrication of nanopaper

**Figure 1.**
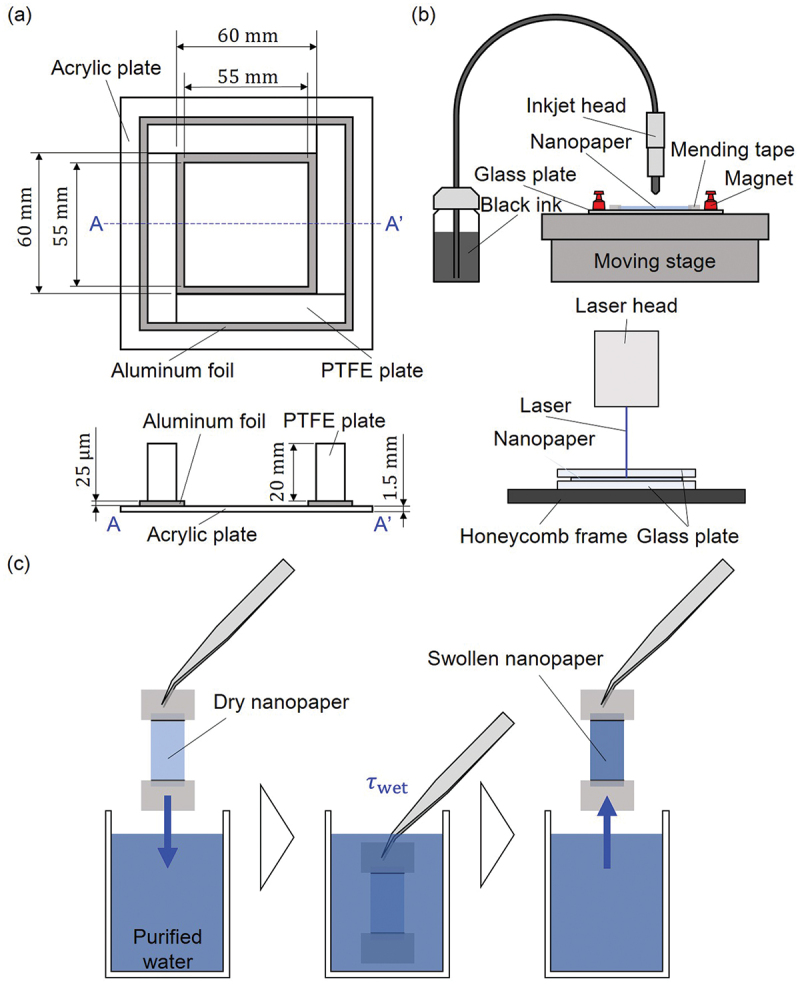
Schematic diagrams for sample fabrication: (a) container to dry aqueous CNF dispersion for nanopaper fabrication, (b) inkjet drawing and laser processing for specimen preparation including kirigami structures, and (c) dipping nanopaper specimen to fabricate CNF hydrogel for a certain wetting time $\tau_{\mathrm{wet}}$.

The so-called nanopaper, i.e., the dry film structure made of CNFs, is fabricated from drying the aqueous dispersion of CNFs. The container to dry the aqueous dispersion consists of bottom wall made of acrylic plate (ACSH-90-90-1.5, MISUMI Corporation, Japan) and side walls made of PTFE (PTFEN-70-20-10, MISUMI Corporation, Japan) as shown in Figure 1(a). Aluminum foil (Myfoil, thickness:12 μm, UACJ Foil Corporation, Japan) was also placed at the peripheries of bottom wall for the ease of detachment of the nanopaper from the container [32]. In this study, the TEMPO-oxidized CNFs (I-2SX, DKS Co., Ltd., Japan) was used for sample fabrication. Originally 2.2 wt% of aqueous CNF dispersion was first placed in a container (A-6 115 mL, MI CHEMICAL, Japan) using a spatula and diluted with purified water (Purified water, Kenei Pharmaceutical Co., Ltd., Japan) to be 0.3 wt% for samples of 15 $g/m^{2}$ and 30 $g/m^{2}$, and 0.6 wt% for those of 0.6 $g/m^{2}$, respectively. The quantities of purified water for dilution were determined by the electronic balance (MS205DU, Mettler-Toledo, LLC) and pipetted (Eppendorf Research® plus Single Channel 1–10 mL, Eppendorf Research® plus Single Channel 100–1000μL, and Eppendorf Research® plus Single Channel 1–10 mL, with pT.I.P.S® Standard/Bulk 0.5–10 mL, Eppendorf, and epT.I.P.S® Reloads 50–1000μL, Eppendorf, Germany). The nominal values of CNF mass $M_{\mathrm{CNF}}$ per unit area of nanopapers for the evaluation of swelling behavior were 15 $g/m^{2}$, 30 $g/m^{2}$, and 50 $g/m^{2}$, where drying process leaves some fraction of CNFs on the side walls. $M_{\mathrm{CNF}}$ was varied by the quantity of CNF dispersion per unit bottom area.

The mixing for dilution was first conducted by combination of rotation of 800 rpm with revolution of 2000 rpm for 5 min for the container with a mixer (AR-100, THINKY CORPORATION), followed by ultrasonic homogenizer (NR-300 M with tip: NR-300 M-MT12, Microtec Co., Ltd.) at 40% of PWM control for 5 min. Another 5 min of rotation at 2200 rpm was applied for degassing the sample after ultrasonic mixing. The sample was then pipetted (M1000E with tip CP1000, Gilson) to the above-mentioned container to dry the sample. Before drying the sample, the degassing with vacuuming at 20 hPa for 10 min was conducted in the chamber (VOM-1000B, Tokyo Rikakikai Co., Ltd., Japan) with a pump (NVP-2100 V, Tokyo Rikakikai Co., Ltd.). The drying process was conducted at 40${\;}^{\circ }$C in another chamber (THR030FA, TOYO ROSHI Co., Ltd.).

**Figure 2.**
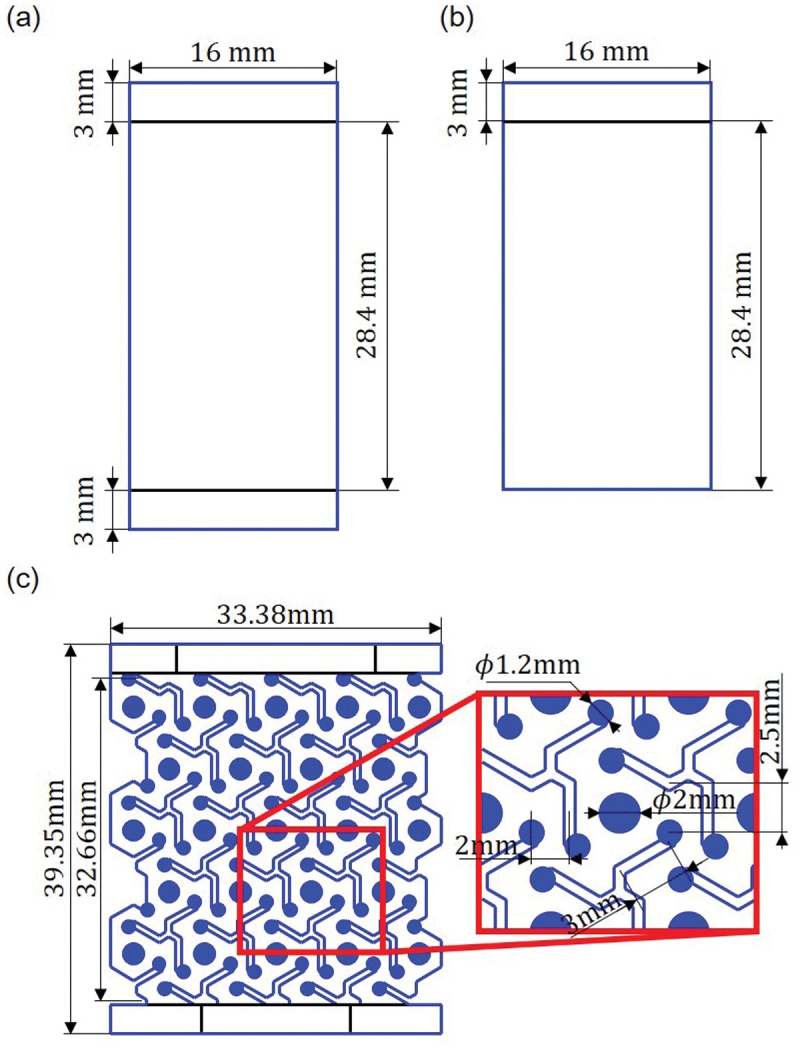
Design of specimens for swelling and tensile experiments: (a) nanopaper without Kirigami, (b) nanopaper for examination of edge boundary conditions, and (c) auxetic Kirigami structure.

The dried up CNF films were manually removed from the acrylic plate, and then cut by laser processing machine (Podea-01, Podea Co., Ltd.) by single scan of the laser head at a speed of 1 mm/s and power of 40% to prepare nanopaper specimen with specified size and shape (cf. Figure 2), where the two black lines of 3-mm distance from the two ends of the specimen are markers for the location of mending tapes two be used for boundary conditions as will be discussed in the later section. Since the nanopapers are transparent, it is nontrivial to process by laser with sufficient precision. Therefore, we first drew lines of black ink (HSM-BK, Seiko Epson Corporation) with inkjet printer (LaboJet-600Basic with a head IJHD-1000, Microjet Corporation) where the laser cutting was to be conducted, which makes higher efficiency of energy conversion of the light to heat for the processing. For the flatness and alignment precision, the aluminum frames were removed in advance of inkjet drawing, and the samples were fixed on the glass plate (S9213, Matsunami Glass Ind., Ltd.) with mending tapes (Figure 1(b)) and placed on the stage of the printer with magnets. The inkjet conditions were tuned as 1st pulse width of 75 μs, frequency of 120 Hz, droplet speed of 6.5 m/s, and dot pitch of 200 μm for line drawing. The sample was placed between glass plates of thickness 2 mm (3-2421-01, AsOne corporation) in the laser processing (Figure 1(b)).

### 2.2. Fabrication of CNF hydrogel

The nanopapers made of CNFs form hydrogels when in contact with liquid water [45]. Therefore, we fabricated the CNF hydrogel specimens by the procedure as shown in Figure 1(c). As schematically shown, mending tapes were sticked from both sides for each end of the specimen (cf. Figure 2(a)). That is, four pieces of mending tape were used for boundary conditions of the two ends. This boundary condition of the specimen was designed for the handling of the specimens by tweezers without damaging them. We also examined the effect of boundary condition assigned by the mending tapes on the swelling behavior by the specimen shown in Figure 2(b) with $M_{\mathrm{CNF}}=15g/m^{2}$. The important parameter to control in the swelling process is the time $\tau_{\mathrm{wet}}$ to dip the dry nanopaper in purified water. We varied $\tau_{\mathrm{wet}}$ to examine the swelling dynamics. The swelling behavior was evaluated by the increase of mass and the size. In the following, specific methodologies of evaluation are described in detail.

### 2.3. Evaluation of swelling dynamics

#### 2.3.1. Evaluation of water content in CNF hydrogel

The mass ratio of water content to the swollen paper can be described as

(1) $$\phi_{F}=\frac{m_{s}-m_{d}}{m_{s}},$$

where $m_{d}$ and $m_{s}$ are masses of dry and swollen paper, respectively. This quantity $\phi_{F}$ defined above indicates the fraction of water in the material that currently contains it. While this is a straightforward definition for ordinary papers, swelling-based gelation behavior of nanopaper leads to $\phi_{F}\approx 1$ for rather short $\tau_{\mathrm{wet}}$ as we will show in Section 3.1. Therefore, we also define the absorption ratio $\phi_{A}$ as the mass ratio of water content to the dry nanopaper as

(2) $$\phi_{A}=\frac{m_{s}-m_{d}}{m_{d}}.$$

**Figure 3.**
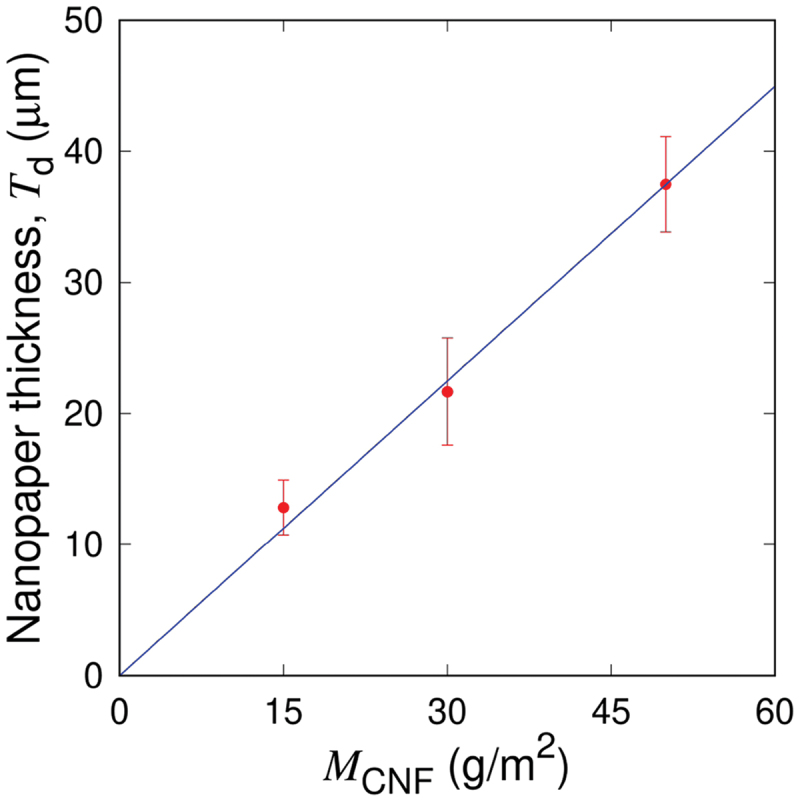
Nanopaper thickness as a function of the mass of CNF per unit area by the fabrication protocol of Figure 1(a). The average values and standard deviations ±S.D. are obtained from at least 30 measurements consisting of 5 locations for each specimen plotted in Figures 7 and 8. The linear scaling of $T_{d}=\gamma M_{\mathrm{CNF}}$ with $\gamma =7.5\times 10^{-4}\;m^{3}/\mathrm{kg}$ holds as shown by the solid line.

The experimental derivation of these values is based on mass $m_{1}$ of a dry entire nanopaper specimen without mending tape, mass $m_{2}$ of a dry entire nanopaper with sticked mending tapes, mass $m_{3}$ of a swollen entire nanopaper with sticked mending tapes, mass $M_{\mathrm{CNF}}$ of CNF per unit area, and the (total projected) area of nanopaper covered with mending tapes. That is, $m_{d}=m_{1}-M_{\mathrm{CNF}}S$ and $m_{s}=m_{3}-(m_{2}-m_{1})-M_{\mathrm{CNF}}S$ where $m_{2}-m_{1}$ is the mass of the mending tapes sticked to the specimen and $M_{\mathrm{CNF}}S$ is the mass of the nanopaper specimen covered with the mending tapes. It should be noted that the parts of nanopaper covered with mending tapes are assumed not to absorb water which will shown to be reasonable. $M_{\mathrm{CNF}}$ ignores the CNFs left on the side walls in the drying process of nanopaper fabrication, which leads to some errors in the relation between the sample thickness but the approximation is in the acceptable range as shown in Table 1 and Figure 3. $m_{1}$, $m_{2}$, and $m_{3}$ were evaluated by an electric balance (MS205DU, Mettler-Toledo, LLC).

**Table 1.** Thickness $T_{d}$ of nanopapers for different $M_{\mathrm{CNF}}$. The average values and standard deviations ±S.D. are obtained from at least 30 measurements consisting of 5 locations for each specimen plotted in Figures 7 and 8.

$M_{\mathrm{CNF}}$ ($g/m^{2}$)	$T_{d}$ (μm)
15	12.8 ± 2.1
30	21.7 ± 4.1
50	37.5 ± 3.6

#### 2.3.2. Evaluation of strains by swelling-based gelation

**Figure 4.**
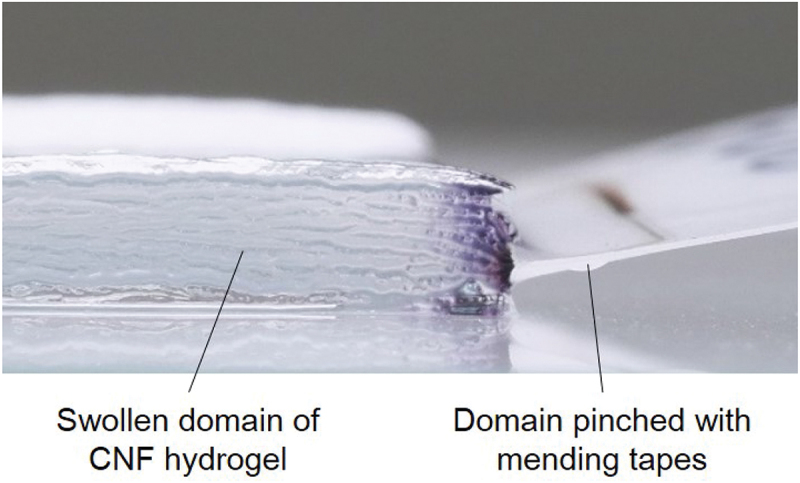
Digital camera image of the edge part of CNF hydrogel formed by swelling nanopaper and subdomain pinched with mending tapes.

**Figure 5.**
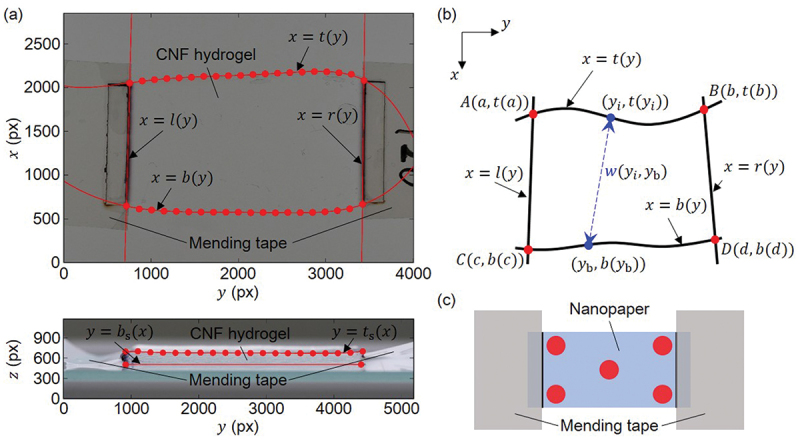
Schematic diagrams for evaluation of swelling dynamics: (a) curve fitting for hydrogel edge lines, (b) width evaluation (cf. Equations (3)–(5)), and (c) locations of thickness evaluation by a micrometer.

As shown in Figure 4, the nanopapers show highly anisotropic swelling behavior when the ends are fixed by mending tapes. Therefore, overall strains in each direction of each specimen due to the swelling were evaluated based on the images of samples placed on a slide glass, captured from horizontal and vertical directions by a digital camera (DC-GX7MK3K and DMC-GX8, Panasonic Corporation) with macro lens (M.ZUIKO DIGITAL ED 60 mm F2.8 Macro, OM Digital Solutions Corporation). Figure 5 schematically shows the methodology to evaluate the strains $\varepsilon_{W}$, $\varepsilon_{L}$, $\varepsilon_{T}$ in the width, longitudinal, and thickness directions, which are defined as nominal values based on the initial state of dry nanopaper specimens. The mean width $w_{s}$ of the CNF hydrogels is defined by

(3) $$w_{s}=\frac{1}{n_{p}}\sum_{i=1}^{n_{p}}w_{m}(y_{i}),$$

(4) $$w_{m}(y_{i})=\underset{y_{b}\in [c,d]}{\min}w(y_{i},y_{b}),$$

(5) $$w(y_{i},y_{b})=\sqrt{{\left(y_{i}-y_{b}\right)}^{2}+{\left(t(y_{i})-b(y_{b})\right)}^{2}},$$

where $n_{p}$ indicates the number of pixels to evaluate the boundary position. The thickness of the hydrogel is evaluated in the similar manner, and the thickness of dry nanopaper samples are evaluated by a micrometer by the mean values based on five locations for each specimen as schematically shown in Figure 5(c). Since it was nontrivial to automatically determine the edge lines between transparent hydrogel and air without missing points, even-spaced 20 points in the longitudinal direction was determined by visual inspection and then fitted by fifth order polynomial function. The straight lines of the boundaries between the glass and hydrogel and those between the hydrogels and mending tapes were determined by the visual inspection of the images. We also evaluated the volumetric strain $\varepsilon_{V}=(V_{s}-V_{d})/V_{d}$ by volumes $V_{s}$ and $V_{d}$ of swollen and original dry samples, respectively. $V_{d}$ is defined as the designed specimen area multiplied by the measured thickness. $V_{s}$ is obtained by the area and thickness evaluated by the procedure mentioned above. Namely, the area is the closed domain defined in Figure 5(a). The mean length of a hydrogel sample for the evaluation of the longitudinal strain $\varepsilon_{L}$ was derived by the evaluated area divided by $w_{s}$.

### 2.4. Design and fabrication of hydrogel Kirigami

We designed auxetic Kirigami structure as shown in Figure 2(c). As we had addressed in our previous study [32], we designed the round edges of the cutting pattern for stress defocusing. In addition, we left some gap width in the cutting lines that is larger than minimum for laser processing to avoid possible sticking of parts during deformation. The precise and complex processing of Kirigami structure for transparent hydrogel that contains large fraction of water is nontrivial. We realized it by the combination of laser processing of the dry film. The anisotropic swelling where swelling in the thickness direction is dominant compared to the in-plane swelling is important for avoiding sticking by vanishing gap of cut lines. The CNF hydrogel specimens with Kirigami structure were fabricated under the condition of $M_{\mathrm{CNF}}=50g/m^{2}$ and $\tau_{\mathrm{wet}}=30$ s. We used sufficiently large container (PS-25, ASVEL Co., Ltd.) in order not to induce unnecessary shear rate of convection when dipping the specimen in water. For comparison, hydrogel specimens without Kirigami structures were also fabricated with $M_{\mathrm{CNF}}=50g/m^{2}$ and $\tau_{\mathrm{wet}}=35$ s. Namely, we tuned $\tau_{\mathrm{wet}}$ to match the hydrogel thickness with the same $M_{\mathrm{CNF}}$.

### 2.5. Evaluation of mechanical characteristics

#### 2.5.1. Specimen setup and cyclic loading test

**Figure 6.**
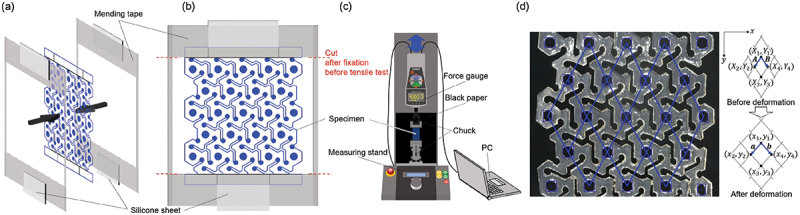
Schematic diagrams of the specimen setup for tensile test and analysis: (a) fixation of the two ends of sample hydrogel with a rectangular frame of mending tape, (b) the front view of specimen before setup on the tensile test system, (c) the tensile testing system consisting on a force gauge and a stand to move it, and (d) schematic of the image analysis for evaluation of the Poisson’s ratio with the actual snapshot of the sample.

Since the specimens of hydrogels were soft, we designed the setup to fix them on the testing system. The dry nanopaper implemented with Kirigami structure (cf. Figure 2(c)) was first pinched with mending tapes to keep the edge sufficiently dry without significant swelling (cf. Figure 6(a)). The mending tape was also spanned to connect two edges of the longitudinal direction so that the end-to-end distance of the specimen is kept as that of dry state. These side parts of the mending tape were cut before starting the tensile test (cf. Figure 6(b)) after setting up at chucks (cf. Figure 6(c)). The parts at which the chucks pinch the specimen is aided with silicone sheets (SR-50, 6-9085-14, AsOne corporation) with a thickness of 0.3 mm with double-sided tapes to prevent the out-of-plane deformation on fixation at the chucks. We defined the two conditions of relaxation time of samples for higher reproducibility. We fixed upper side of the specimen by the chuck (FC-21, IMADA CO., LTD.) of the tensile testing system (EMX-1000 with ZTA-5N, IMADA CO., LTD.) and waited for $\tau_{R1}=5$ min, and then lower side of the specimen was fixed by the chuck (KC-100, IMADA CO., LTD.) and waited for $\tau_{R2}=5$ min before starting the tensile loading. The two sides of mending tapes to keep initial end-to-end distance of the specimen was cut during $\tau_{R2}$ (cf. Figure 6(b)). Starting from the end-to-end distance at the dry state of nanopaper, the Kirigami hydrogel first undergoes the elongation to the maximum strain $\varepsilon_{\max}$ of 0.1 based on on the original length before swelling, i.e. 32.66$\times 10^{-2}$ m (cf. Figure 2(c)). The strain rate was $10^{-2}s^{-1}$. After reaching the maximum strain, the end-to-end distance was restored to the original value. This is the unit cycle of the cyclic loading, and the total number of cyclic loading in a test was assigned as 300. Besides the load-strain relation, we also captured the shape of specimen in time series by digital cameras (DMC-GX8, Panasonic Corporation) with macro lens (M.ZUIKO DIGITAL ED 60 mm F2.8 Macro, OM Digital Solutions Corporation) from two sides. The room temperature was in the range of 24.8–25.1 ${\;}^{\circ }$C and relative humidity was in the range of 35–43%RH.

#### 2.5.2. Evaluation of Poisson’s ratio

The sequential images captured during the cyclic tensile test were analyzed to evaluate the Poisson’s ratio ν. Each image was first processed with the median filter to average and then Sobel filter to detect edges of the specimen. The image was binarized with thresholds of 10, 8, and 15 for Sample 1, 2, and 3 by visual inspection, and then the contour was determined. Since the markers for the evaluation of deformation were circles, we used the roundness criterion defined by the ratio of area of interest to that of a circle with as $4\pi S_{c}/L_{c}^{2}$, where $S_{c}$ and $L_{c}$ are the area and contour line length of the object of interest. From the candidate objects with 3.5mm<L<7.5mm, the roundness was evaluated to distinguish the actual markers with threshold values specifically defined in the range of 0.50 to 0.63 for each sample. In order to avoid misdetection of round edge of the cut lines etc., the outliers of the area of circumscribed rectangles in the detected objects were not employed. This procedure gives the detected marker points as shown in (Figure 6(d)), and thereby the relative position vectors were obtained to evaluate the Poisson’s ratio.

The Poisson’s ratio ν is defined as follows:

(6) $$\nu \equiv -\frac{\varepsilon_{\mathrm{xx}}}{\varepsilon_{\mathrm{yy}}},$$

where $\varepsilon_{\mathrm{yy}}$ is the strain in the tensile/compressive direction and $\varepsilon_{\mathrm{xx}}$ is that in the perpendicular direction, respectively. The deformation gradient tensor F can be described using the strains as follows:

(7) $$F=\left(\begin{array}{cc} \varepsilon_{\mathrm{xx}}+1 & \varepsilon_{\mathrm{xy}}\\ \varepsilon_{\mathrm{yx}} & \varepsilon_{\mathrm{yy}}+1 \end{array}\right),$$

and F relates the vectors before and after deformation shown in Figure 6(d) as follows:

(8) a=FA,

(9) b=FB,

These vectors can be evaluated by the components shown in Figure 6(d) as

(10) $$A\equiv \left(\begin{array}{c} A_{x}\\ A_{y} \end{array}\right)=\frac{1}{2}\left(\begin{array}{c} X_{2}-X_{1}+X_{3}-X_{4}\\ Y_{2}-Y_{1}+Y_{3}-Y_{4} \end{array}\right),$$

(11) $$B\equiv \left(\begin{array}{c} B_{x}\\ B_{y} \end{array}\right)=\frac{1}{2}\left(\begin{array}{c} X_{4}-X_{1}+X_{3}-X_{2}\\ Y_{4}-Y_{1}+Y_{3}-Y_{2} \end{array}\right),$$

(12) $$a\equiv \left(\begin{array}{c} a_{x}\\ a_{y} \end{array}\right)=\frac{1}{2}\left(\begin{array}{c} x_{2}-x_{1}+x_{3}-x_{4}\\ y_{2}-y_{1}+y_{3}-y_{4} \end{array}\right),$$

(13) $$b\equiv \left(\begin{array}{c} b_{x}\\ b_{y} \end{array}\right)=\frac{1}{2}\left(\begin{array}{c} x_{4}-x_{1}+x_{3}-x_{2}\\ y_{4}-y_{1}+y_{3}-y_{2} \end{array}\right),$$

where each of these vectors is evaluated as the mean values of two samples based on the grid [54,55]. Based on the relations Equations (8) and (9), these samples determines F as

(14) $$F=\left(\begin{array}{cc} a_{x} & b_{x}\\ a_{y} & b_{y} \end{array}\right){\left(\begin{array}{cc} A_{x} & B_{x}\\ A_{y} & B_{y} \end{array}\right)}^{-1}$$

(15) $$=\frac{1}{A_{x}B_{y}-B_{x}A_{y}}\left(\begin{array}{cc} a_{x}B_{y}-b_{x}A_{y} & -a_{x}B_{x}+b_{x}A_{x}\\ a_{y}B_{y}-b_{y}A_{y} & -a_{y}B_{x}+b_{y}A_{x} \end{array}\right).$$

The sampled values of Poisson’s ratio can be determined by Equations (6), (7), and (15). 19 circles with a diameter of 2 mm in the center of hexagonal unit in a specimen were tracked (cf. Figure 6(d)). The evaluation of the Poisson’s ratio is based on the ε=0.1, i.e., the state of maximum end-to-end distance, whereas the reference state is the ε=0, i.e., the state of minimum end-to-end distance, in a unit cycle of loading. The Poisson’s ratio was evaluated on condition that all of the 23 circles were successfully detected in the analysis. 4 points at the edges of the specimen were not employed in the evaluation. Therefore, the evaluation ends before the total 300 cycles of the loading.

## 3. Results and discussion

We first discuss the swelling-based gelation behavior of cellulose nanopaper, and then mechanical characteristics of CNF hydrogel Kirigami in comparison with a simple plate-type specimen.

### 3.1. Swelling-based gelation dynamics of cellulose nanopapers

**Figure 7.**
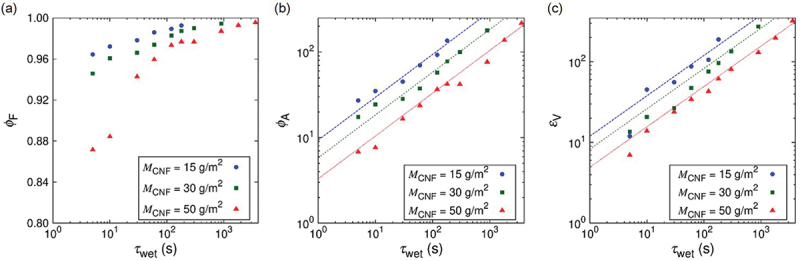
Water absorption and swelling dynamics of cellulose nanopaper to form CNF hydrogels: (a) water content fraction $\phi_{F}$ (cf. Equation (1)), (b) water absorption ratio $\phi_{A}$ (cf. Equation (2)), and (c) volumetric strain $\varepsilon_{V}=(V_{s}-V_{d})/V_{d}$, where $V_{s}$ and $V_{d}$ are volumes of swollen and original states, as a function of wetting time $\tau_{\mathrm{wet}}$. The different points in the figures are obtained by different samples. The coefficients for the fitting curves of $\phi_{A}=C_{A}\tau_{wet}^{0.5}$ are $C_{A}=9.38,5.82$, and 3.30 for $M_{\mathrm{CNF}}=15g/m^{2},30g/m^{2}$, and $50g/m^{2}$, respectively. The coefficients for the fitting curves of $\varepsilon_{V}=C_{V}\tau_{wet}^{0.5}$ are $C_{V}=12.0,8.28$, and 4.95 for $M_{\mathrm{CNF}}=15g/m^{2},30g/m^{2}$, and $50g/m^{2}$, respectively.

**Figure 8.**
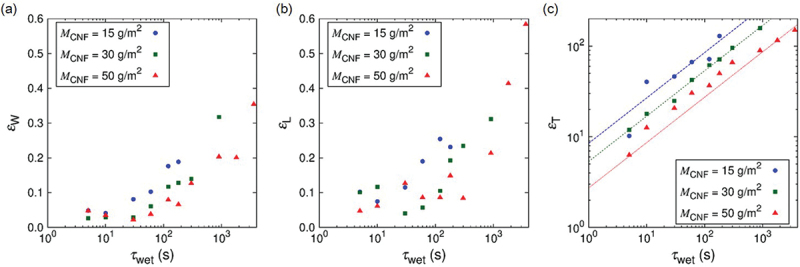
Wetting-time $\tau_{\mathrm{wet}}$ dependence of swelling strains in the specimen (a) width, (b) longitudinal, and (c) thickness directions. The different points in the figures are obtained by different samples. The coefficients for the fitting curves of $\varepsilon_{T}=C_{T}\tau_{wet}^{0.5}$ are $C_{T}=8.49,5.35$, and 2.73 for $M_{\mathrm{CNF}}=15g/m^{2},30g/m^{2}$, and $50g/m^{2}$, respectively.

Figure 7 shows the water content fraction $\phi_{F}$ (cf. Equation (1)), water absorption ratio $\phi_{A}$ (cf. Equation (2)), and volumetric strain $\varepsilon_{V}$ as a function of wetting time $\tau_{\mathrm{wet}}$. The water content fraction $\phi_{F}$ reach 90% within 1 min, water absorption ratio $\phi_{A}$ and volumetric strain $\varepsilon_{V}$ reach $10^{2}$ in $10^{2}$ s for wide range of nanopaper thickness. The fundamental scaling behavior of $\phi_{A}$ and $\varepsilon_{V}$ as a function of $\tau_{\mathrm{wet}}$ is the same regardless of the thickness of the samples. Namely, $\phi_{A}=C_{A}\tau_{wet}^{1/2}$ and $\varepsilon_{V}=C_{V}\tau_{wet}^{1/2}$ are observed. Furthermore, the swelling-based gelation of the nanopapers are highly anisotropic as shown in Figure 8 (cf. Figure 4). Whereas the strains $\varepsilon_{W}$ and $\varepsilon_{L}$ in the width and length directions are within 0.2 for $\tau_{\mathrm{wet}}\;=10^{2}$ s, the strain in the thickness direction approaches $\varepsilon_{T}\;=10^{2}$. Since the swelling is dominated by the strain in the thickness direction, the scaling law of $\varepsilon_{T}=C_{T}\tau_{wet}^{0.5}$ holds. The scaling to the power of 1/2 is mainly attributed to the diffusion dynamics. Based on the consideration of diffusion of water into the hydrogel to results in the swelling behavior, the thickness of the thin plate of hydrogel is predicted to increase in proportional to $\tau_{wet}^{1/2}$ [56].

**Figure 9.**
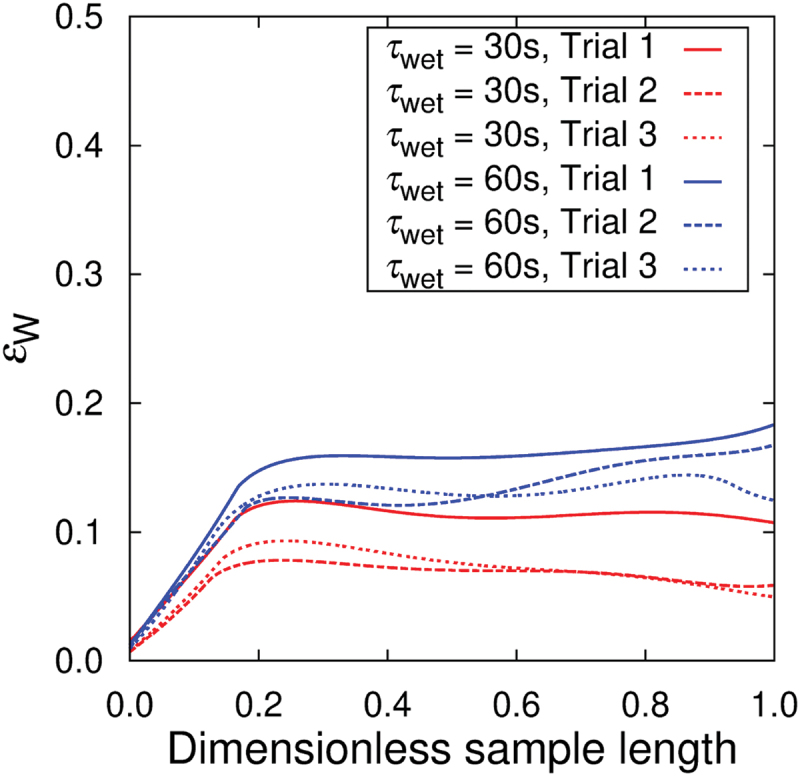
Effect of boundary condition of pinching by mending tapes on the strain profile in the width direction of hydrogel sample with a configuration of Figure 2(b). The sample length is scaled by each of the total length. The side of 0 corresponds to the edge with mending tapes. ‘Trial’ indicates a label for a hydrogel sample.

From more specific point of view, the time $\tau_{\mathrm{wet}}$ necessary for specific values of $\phi_{F}$, $\phi_{A}$, $\varepsilon_{V}$, and $\varepsilon_{T}$ is shorter for thinner nanopapers, which is qualitatively understood from the area-to-volume ratio of the samples. More quantitatively, $C_{A}=9.38,5.82$, and 3.30, $C_{V}=12.0,8.28$, and 4.95, and $C_{T}=8.49,5.35$, and 2.73 for $M_{\mathrm{CNF}}=15g/m^{2},30g/m^{2}$, and $50g/m^{2}$, respectively. In other words, when the nanopaper thickness is 168% and 291% of the case of $M_{\mathrm{CNF}}=15g/m^{2}$ (cf. Table 1), $C_{A}$ was 100/161% and 100/284% and $C_{T}$ was 100/159% and 100/310% of that in the case of $M_{\mathrm{CNF}}=15g/m^{2}$, respectively. We had used this scaling relation to decide $\tau_{\mathrm{wet}}$ (cf. Section 2.4) for the design of simple plate hydrogel specimens to compare with the Kirigami hydrogel specimens. The proportional relation appears to be less clear for strain scaling partly because of the precision of measurements. The origin of less ideal condition is shared in common by the boundary condition to pinch the nanopaper edges (cf. Figure 2(a)) to be less invasive by the treatment with the tweezers (cf. Figure 1(c)). Figure 9 shows the effect of fixed boundary as a function of time. This test was conducted with the boundary condition shown in Figure 2(b). As qualitatively expected, the unfixed end of the specimen is roughly straight in contrast to the fixed end. The influence of the fixed end on the width turned out to be ca. 20% of the total length.

### 3.2. Response to the cyclic loading

**Figure 10.**
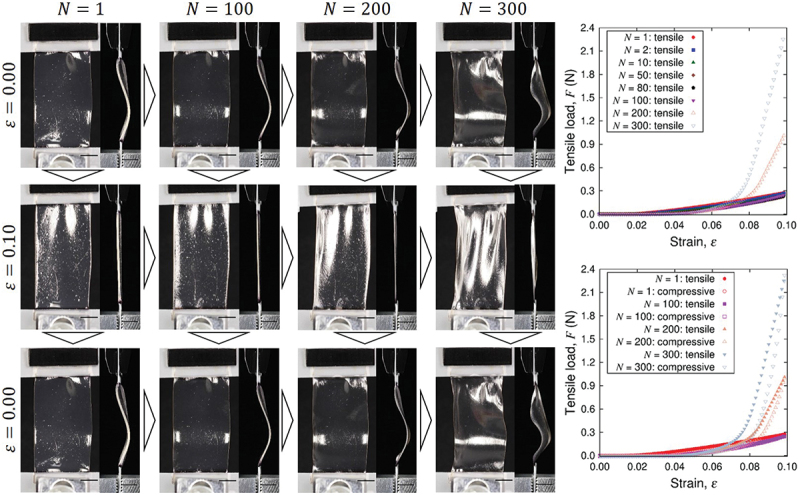
Sequential snapshots of a plate hydrogel sample under cyclic loading test and the obtained tensile load shown as a function of nominal strain ε and the number N of cycles. The scale bars indicate 5 mm. Since the nominal strain is defined by the reference length based on the dry nanopaper state, ε=0 shows significant deflection from N=1.

**Figure 11.**
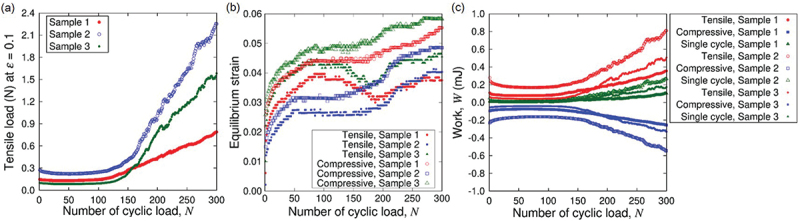
Mechanical response of plate hydrogel samples to the cyclic loading: (a) tensile load at the maximum strain ε=0.1, (b) the strains at which the load changes the sign, and (c) work done on the sample. The results of three sample specimens are shown explicitly for each figure. The sample shown in Figure 10 corresponds to ‘sample 2’ in this figure.

**Figure 12.**
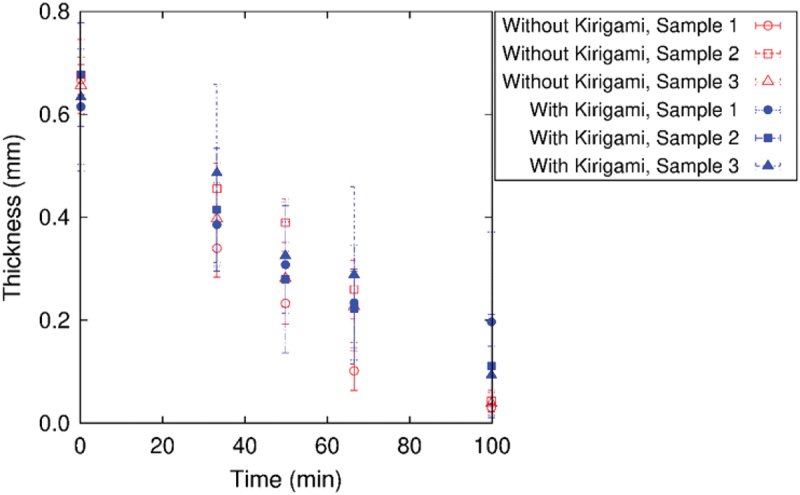
Thickness of CNF hydrogel samples with and without Kirigami structure as a function of time during the cyclic test. The error bars indicate standard deviations from 10 locations of thickness evaluation from the captured images for each sample at ε=0.1. 100 min of time corresponds to 300 cycles of loading in Figure 14, and data points are obtained from the images of N=0,100,150,200, and 300.

Figure 10 shows the sequential snapshots of a simple plate sample under cyclic loading test and the corresponding load-strain relation. The purpose of experiments and analysis of simple plate samples is to characterize the Kirigami design effect. Since the Kirigami sample is of obviously nonuniform shape, we do not define overall stress to represent the sample as a whole here. The initial state of the sample is not straight in the tensile direction but significant deflection in the out-of-plane direction is observed. This is due to the definition of original length (end-to-end distance) based on the dry state of the nanopaper instead of the CNF hydrogel. Nevertheless, the load-strain relation indicates the tensile load from the first cycle of the elongation, and the larger tensile strain ε gives higher tensile load. After a certain number of cyclic loading, the load-strain relation shows substantial deviation from the early stage of the test. This transition occurs around 150 cycles, which is confirmed from Figure 11(a). The tensile load at maximum strain ε=0.1 grows with the increase of cycles thereafter. The difference of shape is not obvious from the observation in the plane normal direction at ε, but it is noticeable at ε=0.1 from the light reflection Figure 10). The difference is related to the drying process, which causes the decrease of sample thickness as shown in Figure 12. This figure also shows sufficient agreement of initial thickness between the plate samples and Kirigami ones.

**Figure 13.**
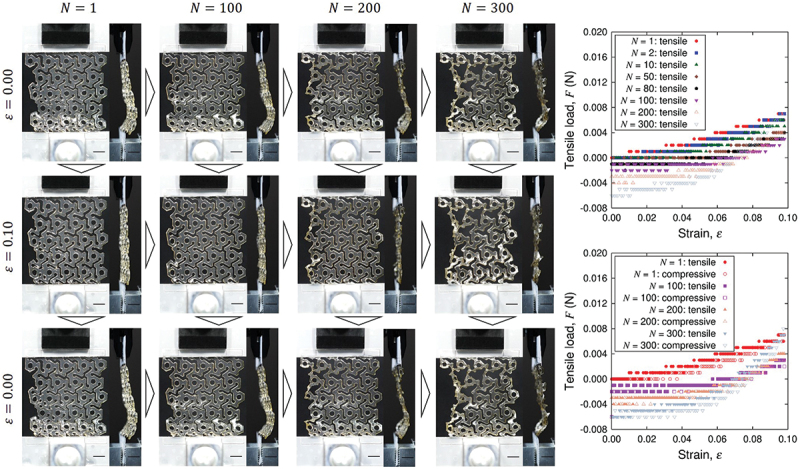
Sequential snapshots of a kirigami hydrogel sample under cyclic loading test and the obtained tensile load shown as a function of nominal strain ε and the number N of cycles. The scale bars indicate 5 mm. Since the nominal strain is defined by the reference length based on the dry nanopaper state, ε=0 shows significant deflection from N=1.

Now we look into the hydrogel Kirigami samples. The load-strain relation in Figure 13 shows the immediate tensile load from the first cycle of the elongation without compressive load. In common with the case of simple plate. On the other hand, the maximum load in a loading cycle is substantially smaller than plate samples. This is partly because of the smaller characteristic width of the samples for Kirigami although the span width of an entire sample in the direction perpendicular to the tensile direction is larger (cf. Figure 2). However, the fundamental qualitative difference is the Kirigami structure that causes elongation at the whole sample scale is realized by the deformation that combines bending with rotation of hexagonal elements. The maximum nominal strain ε=0.1 is sufficient to show the deviation of load-strain relation in the forward (tensile) and backward (compressive) from the first cycle. As the number N of the cycle substantially increases, the cyclic loading rather induces compressive load (i.e. negative value in the tensile load in the figure). The magnitude of compressive load at N=300 is in the same order as that of tensile load at N=1. The sample responds to the elongation of ε=0.1 to show in-plane deformation as expected at least up to N=100. The drying process causes contraction of the sample to end up out-of-plane deformation at N=300 even at ε=0. The significant contraction due to drying is also observed from the increased gap of the periodic units in the sample. The mean thicknesses of the three samples at N=1,100,150,200, and 300 were 0.64, 0.43, 0.30, 0.25, and 0.13 mm, respectively, which is summarized in Figure 12.

**Figure 14.**
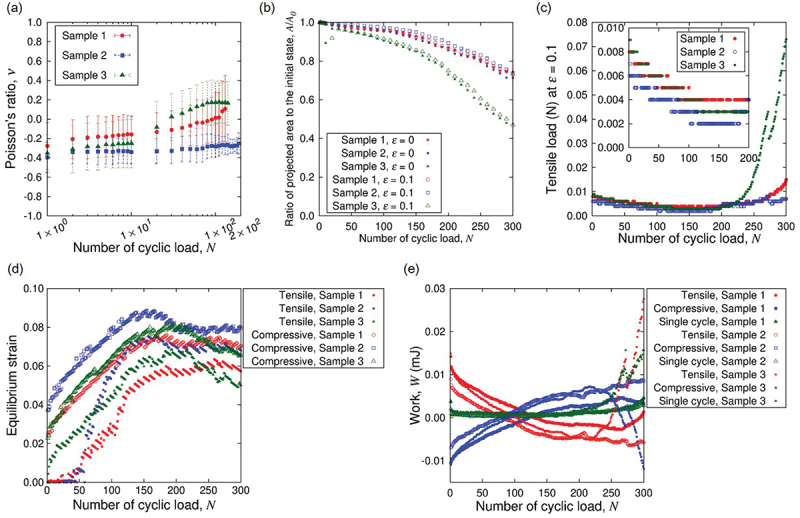
Mechanical response of Kirigami hydrogel samples to the cyclic loading: (a) Poisson’s ratio ν, (b) the ratio of the projected area to the initial value, (c) tensile load at the maximum strain ε=0.1, (d) the strains at which the load changes the sign, and (e) work done on the sample. The results of three sample specimens are shown explicitly for each figure. The error bars in (a) indicates the standard deviation in a specimen obtained from 10 values consisting of different sets of 4 relative position vectors (cf. Figure 6). The sample shown in Figure 13 corresponds to ‘sample 2’ in this figure.

The in-plane deformation of the sample supports the auxetic behavior as shown in Figure 14(a). Depending on the difference in the initial configuration of the sample due to the swelling-induced buckling at N=0, the auxetic behavior can diminish at $N∼10^{2}$. However, the sample can resist the out-of-plane deformation in the presence of initial buckling state as shown in Figure 13 that corresponds to ‘Sample 2’ in Figure 14. In this sample, the auxetic characteristics is preserved until N=200. Since the in-plane deformation is important in the auxetic behavior of the present design, we evaluated the ratio of projected area A to the initial value $A_{0}$ as a function of N as shown in Figure 14(b). The sample with shorter endurance of auxetic behavior (i.e. ‘Sample 3’) shows smaller $A/A_{0}$ compared to other samples. In particular, the rate of decrease in $A/A_{0}$ increases at N≈150−200. Since the variation of humidity is small as mentioned in Section 2.5.1, the significant deviation of $A/A_{0}$ for Sample 3 is attributed to the difference in the deformation (due to initial configuration). This interpretation is supported by the high reproducibility of $A/A_{0}$ for Sample 1 and Sample 2.

The N dependence of geometric quantities ν and $A/A_{0}$ indicates the plastic deformation in response to the cyclic loading. This is also confirmed from the tensile load at the maximum strain ε=0.1 as a function of N as shown in Figure 14(c). The tensile load at ε=0.1 decreases with increasing N until ca. N=150. This is interpreted as the adaptive response of the sample to the repeated elongation. The increase of the load for N>200 is mainly attributed to the change of state due to substantial drying (cf. Figure 12), while the drastic increase of the load for Sample 3 is combined with the transition of deformation state. We examined the drying rate $Q_{\mathrm{dry}}$ of the sample by monitoring the load as a function of time without change of end-to-end distance. In the cyclic loading test, the strain rate $10^{-2}s^{-1}$ with the maximum strain ε=0.1 corresponds to $\tau_{\mathrm{cycle}}=20$ s for a single cycle of elongation and contraction, and 300 cycles corresponds to $10^{2}$ min. The rate $Q_{\mathrm{dry}}$ was roughly linear during this period in the experiment with the constant end-to-end distance at the initial value, and the fitting resulted in $Q_{\mathrm{dry}}\approx 1.1\times 10^{-7}$ kg/s. The load decrease due to the mass decrease of the sample by water evaporation for N=150 amounts to $3.2\times 10^{-3}$ N, which is substantially smaller than the observed total decrease of the tensile load at ε=0.1 at N=150. Thus, the tensile load indeed decreased in mechanical response to the cyclic loading. This adaptive plasticity is much less clear for simple plate samples (cf. Figure 11(a)). In other words, the adaptive plasticity is enhanced by the Kirigami design. Furthermore, the flexibility of Kirigami structure is also manifested in the response to drying. The speed of thickness decrease is roughly the same between the Kirigami and simple plate, but the drying-induced loading increase of Kirigami appears later (cf. Figure 14(c)) than the simple plate (cf. Figure 11(a)).

Since the load can be compressive in a single cycle of loading for large N (cf. Figure 13) and yet the load was always tensile at the maximum strain (cf. Figure 14(c)), it is worth look into the equilibrium strain at which the loading vanishes. To be more precise, we examined the strain at which the load changes the sign. This change of sign can take place twice in a single cycle of loading, i.e. from tensile to compressive and vice versa. The results from different samples shown in Figure 14(d) clearly indicate the common features. The response of samples to the cyclic loading consists of a transient period of increase in the equilibrium strain with N and the plateau after N≈150. The transient rise of equilibrium strain is an adaptive response to the cyclic loading from initial condition, and the plateau suggests that the samples reached the asymptotic state corresponding to the cyclic loading. The equilibrium strain is larger in the compressive process compared to the tensile one regardless of N or samples. This hysteresis suggests the existence of plastic deformation from N=1. Even after the samples reached the asymptotic states, this hysteresis persists. The equilibrium strains of simple plate specimens are nontrivial to read the common feature except for the initial rapid increase with increasing N(cf. Figure 11(b)). The strong sample dependence is partly attributed to the nonuniform strain distribution induced in the drying process.

If the deformation were purely plastic, it may well be regarded as a simple plasticity without hardening but rather softening until the drying effect prevails (cf. Figure 14(c)). Therefore, we examine the elastic component of the response to characterize our proposed mechanical metamaterial. When the specimen is elastically elongated, the external work is done on the specimen. The work $W_{\mathrm{tensile}}$ necessary for the elongation ΔL from initial length to the maximum strain $\varepsilon_{\max}$ with the force F(l) as a function of elongation l is described as

(16) $$W_{\mathrm{tensile}}=\int_{0}^{\Delta L}F(l)\mathrm{dl}.$$

Similarly, the work necessary for the compression is described as

(17) $$W_{\mathrm{compressive}}=\int_{\Delta L}^{0}F(l)\mathrm{dl}.$$

The effective work W of a single cycle of loading is then

(18) $$W=W_{\mathrm{tensile}}+W_{\mathrm{compressive}}$$

If the deformation of specimen is purely elastic with a simply positive Young’s modulus, the backward process of contraction results in the release of energy from the specimen with the same amount as the work in the forward process, i.e. W=0. When the process involves the dissipation of energy or some other energy exchange due to the drying process during the tensile test, there is a difference of energy exchange between the forward and backward process and/or W depends on the number N of cyclic loading.

Figure 14(e) shows the exchange of energy between the sample and the external loading system. The sign is defined as W>0 when the work was necessary to deform the sample. When $N\ll 10^{2}$, the work is necessary to elongate the sample but the work is mostly recovered in the backward process to restore the initial end-to-end distance. The irreversible work or dissipated energy is substantially smaller than the necessary work to the tensile process. The sign of necessary work changes for $N>10^{2}$. Namely, it is necessary to apply work on the sample in the compressive process and it is mostly recovered in the tensile process. This feature is maintained for N>150 without drastic transition until the final stage of the test. In fact, this is characteristic to the design of this Kirigami in comparison to the simple plate. Figure 11(c) shows that the reversal of work sign does not occur in the simple plate samples but the necessary work just grows for N>150. In either case, the irreversible work is substantially small compared to the storing and release of elastic energy in the most part of the cyclic loading test. Thus, the system as a whole responds to the cyclic loading rather elastically from energetic viewpoint.

## 4. Conclusions

We have developed mechanical metamaterial made of hydrogel from CNFs implemented with Kirigami structure, and characterized its flexibility in terms of adaptive response. The fabrication of Kirigami structure is based on the highly anisotropic swelling-based gelation where most of the volume increase from the dry cellulose nanopaper originates from the increase in the thickness. The scaling of mass increases as a function of wetting duration is a power law with an exponent of 1/2. This fabrication process can also be useful in the situation of application or usage, where preservation of hydrogel state is nontrivial due to the drying. The fabricated hydrogel metamaterial shows auxetic behavior by the Kirigami design. The negative Poisson’s ratio is kept for a hundred cycles of tensile loading. On the other hand, the strain at which the load changes the sign shows transient plastic response to ca. 150 cycles and then reach a plateau. The tensile load at the maximum strain decreases in this 150 cycles. This adaptive response is attained while keeping the auxeticity, which is important from the viewpoint of multifunctionality as well. The applied deformation is mainly elastic from the energetic viewpoint, but the energy exchange in a cycle approaches an asymptotic state due to plastic deformation. This adaptive plasticity in combination with elastic response and plateau after transient response is important in the design of future intelligent materials based on their softness.
